# Antioxidant activity of *Cinnamomum cassia* extract and quality of raw chicken patties added with *C. cassia* powder and *Pleurotus sajor-caju* powder as functional ingredients during storage

**DOI:** 10.5713/ab.21.0444

**Published:** 2022-03-02

**Authors:** Kanita Galih Julia Rakasivi, Koo Bok Chin

**Affiliations:** 1 Department of Animal Science, Chonnam National University, Gwangju 61186, Korea

**Keywords:** Cinnamon, Lipid Oxidation, Oyster Mushroom, Raw Chicken Patty

## Abstract

**Objective:**

The aim of this study was to investigate antioxidant activities of cinnamon (*Cinnamomum cassia*) extracts (extracted with different solvents) at various concentrations and to determine product quality of raw chicken patties added with different levels of cinnamon powder (CP) and oyster mushroon powder (OMP) during storage.

**Methods:**

After cinnamon was made into oven dried CP and extracted with water and different levels (50%, 80%, and 100%) of ethanol, antioxidant activities of these extracts were determined. CP and OMP were combined at different levels and added to raw chicken patties. Physicochemical properties and microbial counts were measured during refrigerated storage.

**Results:**

Cinnamon ethanol (80%) extract showed the highest (p<0.05) by 2,2-diphenyl-1picrylhydrazyl-radical scavenging activity and reducing power. Cinnamon water extract (CWE) had the highest iron chelating ability (p<0.05), while CP 100% ethanol extract had the highest content of total phenolic compound. Then, CP and OMP were applied to chicken patties at different levels (0.1% to 0.2%). After the addition of CPs, pH, L* (lightness), 2-thiobarbituric acid reactive substance, and volatile basic nitrogen values were decreased, whereas a* (redness) and b* (yellowness) values were increased. Microbial counts of total bacteria and *Enterobacteriaceace* were decreased with the addition of CP 0.2% regardless of the OMP level.

**Conclusion:**

The addition of CP in combination with OMP can increase the shelf-life of chicken patties during storage.

## INTRODUCTION

Antioxidants play an important role in preventing tissue damage by decreasing the production of free radicals, by scavenging them, or by promoting their decomposition. Synthetic antioxidants such as butylated hydroxytoluene (BHT) and butylated hydroxyanisole (BHA) have been used to maintain the quality of fully cooked food products. However, they might impose health risks to human. Thus, consumers demand for the use of natural antioxidants instead of synthetic antioxidants in the food industry to have safe food [1]. Plants and vegetables are known to be natural sources of antioxidants to combat oxidative instability of lipids and proteins in meat and meat products [2].

Extraction is an important procedure to obtain natural antioxidants from fruits and plants. Many extraction factors such as solvent type, solvent concentration, extraction temperature, and extraction time can affect the extraction efficiency [3]. Solvent extraction is the most common method used to extract natural oxidants from plants. However, the extraction yield is affected by the extraction solvent, the extraction temperature, the extraction time, and the chemical nature of the sample. The solvent used and the chemical nature of the sample are the two most important factors affecting the extraction [4]. Lipid peroxidation is a process that involves the reaction of polyunsaturated fatty acids (PUFAs) in phospholipids of cellular membranes with oxygen to produce lipid hydroperoxides (ROOH). This reaction might occur via a free radical chain mechanism which starts by abstracting a hydrogen atom from a PUFA by a reactive free radical, followed by propagation [5].

*Cinnamomum cassia* bark contains seven aromatic compounds: lyoniresinol 3α-O-β-D-glucopyranoside, 3,4,5-trimethoxyphenol β-D-apiofuranosyl-(1→6)-β-D glucopyranoside, (±)-syringaresmol, two epicatechin derivatives, and two cinnamic aldehyde cyclic glycerol 1,3-acetals [6]. Muchuweti et al [7] evaluated contents of phenolic compounds in several spices such as baby leaves, rosemary, sage, majoram, and organo cinnamon and found that *Cinnamonum zeylanicum* (cinnamon) contains 13.66 mg gallic acid equivalents (GAE)/g of polyphenolic compounds and phenolic compounds such as vannilic acid, caffeic acid, and ferulic acid. Murcia et al [8] compared antioxidant properties of seven dessert spices (anise, cinnamon, ginger, licorice, mint, nutmeg, and vanilla) and synthetic food antioxidants such as BHA, BHT, and propyl gallate and found that mint and cinnamon had higher antioxidant activities than other spices analyzed. In addition, irradiation as a decontamination method at up to 10 kGy did not affect antioxidant activities of these spices.

*Pleurotus sajor-caju* (oyster mushroom) is rich in nutritional components such as protein, carbohydrates, crude fiber, and minerals including Ca, Fe, Mg, Na, K, and P with a very lower level of fat. This species has significant amounts of antioxidants such as phenols, ascorbic acid (AA), and flavonoids with antibacterial activities. These functional properties of oyster mushroom might prevent various deficiencies, diseases, and malnutrition [9]. Although many studies have reported antioxidant and antimicrobial activities of individual antioxidants from various spices, studies on the synergistic effect of a combination of two or three antioxidant from natural resources have not been reported yet. Effects of a combination of cinnamon and oyster mushroom powders (OMPs) on meat products and their quality characteristics are not well understood yet. Thus, the objectives of this study were: i) to prepare cinnamon and OMPs with oven-drying methods for easy handling and longer shelf-life; ii) to determine antioxidant activities of cinnamon using different extraction methods; iii) to investigate effects of the addition of a combination of cinnamon and oyster mushroom at different levels on quality characteristics of raw chicken patties.

## MATERIALS AND METHODS

### Preparation of cinnamon extract

#### Cinnamon powder

*Cinnamomum cassia* (Cinnamon bark) was purchased from a local market. Cinnamon bark was dried by oven drying at 60°C until a constant weight was reached. It was then ground using an Ultra-Power mixer (Hanil, Gwangju, Korea). The cinnamon powder (CP) was then sieved using a 500 μm sieve and kept at −20°C until further use.

#### Cinnamon water extract

CP was weighed 10 g and mixed with 200 mL of double distilled (dd)-water. The mixture was homogenized for 5 min and centrifuged at 3,000 rpm for 5 min. After filtering using a filter paper (Whatman no. 41), the extract was kept in a freezer at −70°C for 4 hours. The CP water extract was then placed in a freeze-dryer (Ilshin Lab. Co. Ltd, Seoul, Korea) at −54°C for four days.

#### Cinnamon ethanol extract

Approximately 30 g of CP was weighed and mixed with 600 mL of edible ethanol (50%, 80%, and 100% ethanol). Samples were then stirred overnight at room temperature until powders were completely dissolved by the solvent. The liquid extract was filtered using a filter paper (Whatman no. 41). Extraction solvent was then evaporated using a rotary evaporator (Rotavapor 110; Hwashin Science, Seoul, Korea) completely. Ethanol extracts were then placed in a freezer at −70°C until fully frozen. They were then dried in a freeze dryer for 4 days to obtaine freeze-dried CP ethanol extracts.

### Antioxidant activity analysis of cinnamon

#### 2,2-Diphenyl-1picrylhydrazyl-radical scavenging activity

The antioxidant activity (free radical scavenging activity [RSA]) was determined using a modified method of Huang et al [10]. Briefly, 1 mL solution of each treatment (0.1% to 1.0% in dd-water) and AA as reference were mixed with methanolic 2,2-diphenyl-1picrylhydrazyl (DPPH) (0.2 mM) on a vortex and then placed in a dark room for 30 min. The absorbance of the sample was read using a UV Spectrophotometer (UV 1601; Shimadzu, Kyoto, Japan) at 517 nm.

### Ferrous iron chelating ability

Ferrous iron chelating ability (ICA) was measured using the method described by Le et al [11]. Briefly, 0.5 mL of sample (0.1% to 1.0% in dd-water) was mixed with 0.1 mL of ferrous chloride reagent, 0.5 mL ethylenediamine tetraacetic acid (EDTA), and methanol (0.9 mL). After 5 min, 0.1 mL of ferrozine (5 mM) was added and the sample was held at room temperature for 10 min. The ferrous ICA (%) was determined by measuring the absorbance of the Fe^2+^ ferrozine complex at 562 nm. The EDTA was used as a positive control.

### Ferric reducing power

The ferric reducing power (RP) reagent was prepared as described by Huang et al [10]. The reagent was mixed with 2.5 mL of sample (0.1% to 1.0% in dd-water). After 20 min of incubation at 50°C, 2.5 mL of trichloroacetic acid (TCA 10%) was added and the mixture was centrifuged at 1,500 rpm for 10 min. Subsequently, the upper layer (2.5 mL) was taken and added with 2.5 mL of dd-water and 0.5 mL of ferric acid chloride (0.001%). Reducing power was then measured by reading the absorbance at 700 nm.

### Total phenolic compounds

Cinnamon sample (0.1 g) was mixed with 10 mL of dd-water. Then 0.1 mL of this mixture was mixed with dd-water (2.8 mL), 2% Na_2_CO_3_ (2 mL), and 50% Folin-Ciocalteau reagent (0.1 mL) prior for vortexing. After 30 min of incubation at room temperature, the absorbance of the mixture was measured at 750 nm using a spectrophotometer (UV-1601; Shimadzu, Japan). Gallic acid was used as a standard (0 to 200 mg/L). Total phenolic compounds are expressed as GAE/100 g dried powder [12].

### Manufacture of raw chicken patties

Fresh chicken breasts were obtained from a wholesale meat market in Gwangju, South Korea. They were ground using a grinder (M-12s; Fujee Plant, Busan, Korea). Ground chicken breast, sodium chloride, and additional ingredients were mixed for 1 min, ground in emulsifier, weighed into approximately 60 g, and formed into individual patties. These patties were placed on a polystyrene plate and held inside a refrigerator at 4°C±1°C during 14 days of refrigerated storage [13]. The following groups were included: control (CTL), 0.1% (w/w) ascorbic acid (REF), 0.1% CP + 0.1% OMP (C1M1), 0.1% CM + 0.2% OMP (C1M2), 0.2% CM + 0.1% OMP (C2M1), and 0.2% CP + 0.2% (OMP) (C2M2) (Table 1).

### pH and color values

The pH values of raw chicken patties were determined using a pH meter (Mettle-Toledo, Schwarzenbach, Switzerland) for five different locations per sample. Mean values of triplicate samples were calculated to obtain an average pH value. The color of raw chicken patty was determined with a Minolta color reader (Model # CR-10; Minolta, Tokyo, Japan). Results are presented as lightness (L*), redness (a*), and yellowness (b*). Measurement was performed for the surface at six different locations per patty sample. Mean color values of L*, a*, and b* were analyzed to obtain average colorimetric value.

### 2-Thiobarbituric acid reactive substance

Lipid oxidation of chicken patties during storage was determined by measuring 2-thiobarbituric acid reactive substance (TBARS) [14]. Patty samples were ground, weighed (about 2 g), and mixed with 3 mL of 2.5% thiobarbituric acid (TBA) reagent and 17 mL of 1% TCA in tubes with caps. Tubes were placed in a water bath at 90°C for 30 min. The supernatant of each solution was then mixed with 5 mL chloroform and centrifuged at 2,000×rpm for 5 min (VS-5000 N; Vision Scientific Co. Ltd., Bucheon, Korea). Each supernatant was added with approximately 3 mL of petroleum ether and centrifuged at 2,000×rpm for 10 min. Finally, clear solutions were analyzed with a spectrophotometer (UV-1601; Shimadzu, Japan) at wavelength of 532 nm. TBA value was expressed as mg of malondialdehyde (MDA) per kg meat sample.

### Microbial counts

For microbial counts, 10 g of homogenized sample was mixed with 90 mL of sterilized water using a stomacher lab blender. Serial dilutions were then made. After that, about 0.1 mL of each diluted sample was inoculated and spread onto the surface of violet red bile agar and plate count agar medium. Plates were then incubated at 37°C for 24 to 48 hours. All plates were examined visually to count the number of colonies. Microbial colonies were counted and expressed as log 10 colony forming units/g chicken meat.

### Volatile basic nitrogen

Volatile basic nitrogen (VBN) values (mg %) of chicken patties were measured using the Conway method [15] with slight modifications. Briefly, approximately 1 g of each patty sample was homogenized with 9 mL of dd-water using a homogenizer (S25N-18G; IKA, Staufen, Germany) for 1 min at 11,000 rpm and filtered with a Whatman No. 1 filter paper. Approximately 1 mL of the filtrate was transferred to a Conway dish to react with 1 mL of saturated potassium carbonate (K_2_CO_3_) solution at 37°C for 120 min. The incubated solution was then titrated with 0.01 N HCl. VBN value was expressed as mg %.

### Statistical analysis

All experiments were carried out in triplicate. Data were analyzed using one-way (experiment 1) or two-way (experiment 2) analysis of variance. In experiment 2, if the interaction between two factors was significant (p<0.05), then data were separated by storage time within a treatment or by treatment within a storage time. If the interaction was not significant (p>0.05), then data were pooled by treatment or storage time. Duncan’s multiple range test was used to determine significant differences at 5% level (p<0.05) using SPSS 21.0 program for Windows.

## RESULTS AND DISCUSSION

### Experiment 1. Antioxidant activity of cinnamon as affected by different solvent based on DPPH-RSA assay

DPPH assay has been used to estimate antioxidant activities of various food products. DPPH radical, a very stable nitrogen-centered radical, can be used to determine the free radical scavenging ability, which is related to their antioxidant activity. This method is based on the spectrophotometric measurement of DPPH concentration changes resulting from DPPH reaction with an antioxidant [16]. As shown in Table 2, the positive control (AA) had the highest value of DPPH-RSA (p<0.05). Cinnamon powder and CEE 80 (Cinnamon ethanol extract by 80% ethanol) showed higher DPPH-RSA than other treatments (p<0.05). Radical scavenging activities of CP and CEE 80 ranged from 48.1% to 68.8% and from 44.5% to 59.1%, respectively. The DPPH-RSA value was gradually increased with increasing concentration of CP. Mathew and Abraham [17] have reported that the antioxidant activity (RSA) of CP extracted with methanol at 6.25 to 50 μg/mL is increased with increasing concentration. Manchini-Filho et al [18] have reported that phenolic compounds in CP extracts are active components responsible for the antioxidant activity of ethanol-water extracts of CP. In the present study, the antioxidant activity (RSA) of CP was higher with ethanol extraction, particularly with 80% ethanol. Combination of ethanol and water solvent might be suitable for extracting some bioactive compounds with a broad range of polarity. Truong et al [19] have recently found that different extraction solvents with different polarity caused wide variations in the level of bioactive compounds found in the CP extract.

### Iron chelating ability

Table 2 shows results of ICA of EDTA, CP, and CP ethanol extract. EDTA as a positive control exhibited the strongest ICA (p<0.05) of 95.8% to 98.6%. The order of ICA in this study was: EDTA>CEE 0>CEE 100>CEE 80>CEE 50>CP (ICA ranges: 95.8% to 98.6%, 21.2% to 83.1%, 15.9% to 53.2%, 19.1% to 39.5%, and 23.5% to 31.9%, respectively). CP alone had the lowest ICA (6.77% to 22.5%). CEE 0 showed higher (p<0.05) ICA than CP and CP ethanol extract. ICA of CP water extract (CEE0) was comparable to that of EDTA. This indicates that the chelating compound in CP might be more soluble in water than in ethanol. These results were similar to a previous study of Cuong and Chin [20]. They reported that water extract of *Cudrania tricuspidata* (CT) leaves had higher chelating activity than methanol and ethanol extracts. In addition, ICA was increased with increasing CP concentration (0.1% to 1.0%) (p<0.05).

### Reducing power

As shown in Table 2, solvent used for extraction affected the RP of CP and CP ethanol extract (p<0.05). However, extracts with different solvent at different concentrations had almost similar values. RP increased with increasing CP concentration (p<0.05). Many studies have indicated that antioxidant activity is related to the development of reductones known to react with certain precursors of peroxide and act as terminators of free radical chain reactions [21]. Ascorbic acid (AA) was used as a positive standard. RP showed the following order: AA>CEE 80>CEE 50>CEE 100>CEE 0>CP. This result indicated that CP ethanol extracts had higher reducing potential than CP and CP water extract. Among different concentrations of ethanol used for extraction, 80% ethanol was the most effective one as CEE 80 showed the highest RP. These results were similar to those of Varalakshmi et al [22]. They reported that RP of aqueous extract of CP was lower than that of methanol or chloroform extract of CP. They also reported that bark extracts functioned as an electron donor which reacted with free radicals to convert them to more stable products, resulting in the termination of radical chain reaction. Different types of solvent can affect the extractability of antioxidants. Water could dissolve alkaloid and glycoside compounds. However, ethanol was effective for extracting sterol, flavonoid, phenolic, and alkaloids [23]. Another study has observed that RP of CP extracted by methanol is the highest, followed by CPs of ethanol and water extracts [24]. Kamleshiya et al [25] reported that methanolic extracts of spices had higher RP than aqueous extracts of spices, with methanolic extracts of *Cinnamonum cassia* (150 to 200 μg/mL) and *Piper nigrum* (200 to 250 μg/mL) showing significant activities (>50%).

### Total phenolic contents

Total phenolic contents were determined with a spectrometric method according to the Folin-Ciocalteu phenol method. They are expressed as GAE. Phenolics compounds such as flavonoids and phenolic acid possess various biological activities which might be related to their antioxidant activity. As shown in Table 3, total phenolic content in ethanol extract of CP was higher (p<0.05) than that in CP water extract. Total phenolic contents ranged from 11.77 to 19.73 mg GAE/g, showing the following order: CEE 80>CEE 50>CEE 100> CWE>CP. However, total phenolic content showed no significant difference among different ethanol extracts. Dvorackova et al [26] have shown that a binary solvent system is more effective than a single solvent system, depending on their relative polarity. The optimum solvent percentage was about 60% (v/v) ethanol at a ratio of 1:20 with cinnamon sample and the optimal extraction temperature and time were 50°C and 90 min, respectively. Abeyeskera et al [27] have found that cinnamon (*Cinnamomum zeylanicum Blume*) barks extracted with ethanol have 44.57±0.51 mg GAE/g of total phenolic compound. *Cinnamomum zeylanicum* (*Ceylon cinnamon*) leaf and bark extracts had higher antioxidant activities than extracts of other *Cinnamomum* species such as *C. cassia, C. tamala*, and *C. verum*. Another study has also found that the range of total phenolic compounds in extracts of spices including cinnamon (*Cinnamomum zeylanicum*) was 3.53 to 58.25 mg GAE/g with an average value of 19.9 mg GAE/g. The highest level of total phenolic compounds was observed in galangal, whereas the lowest value was found in white pepper as reported by Lu et al [28]. Six phenolic compounds (catechin, *p*-coumaric, vannilic acid, caffeic acid, ferulic acid, and protocatechuic acid) have been found in CP extracted with subcritical water [29].

Ethanol extracts of freeze-dried CP showed higher levels of phenolic compounds than those of oven-dried CP. Therefore, freeze-drying can enhance the extractability of phenolic compounds since ice crystals can form within the sample matrix and rapture the cell structure, which allows cellular components and solvent to come out as reported by Nicoli et al [30]. Furthermore, the loss of phenolic compounds in CP by oven drying at higher temperature (>88°C) was higher than that by freeze drying. However, thermal processing, sometimes, released more phenolic acids from the breakdown of cellular components due to the heat processing, resulting in accumulation of more antioxidants. During the drying processes, oxidative enzymes such as polyphenol oxidase and peroxidase were deactivated, leading to avoid the loss of phenolic compounds as reported by Dewanto et al [31]. Based on the results of the antioxidant activities, data were not consistent. For example, CEE 80 was highest in DPPH, whereas CEE100 was lowest in total plate counts (TPC) in this stduy, which was similar trend to those of RP. Unlike these data, the iron chelating ability was highest in the water extration (CWE) rather than ethanol extraction (CEE), since the CP might be soluble in water rather than ethanol. In TPC, the CEE 80 was better than the CEE 100. The analysis of anitoxidant activities were not similar trend in this study due to the different mechanism of the various antioxidant measurements as reported by Goulas and Manganaris [32].

### Experiment 2. Physicochemical and textural properties of chicken patties including pH and color value

Table 4 shows pH values of raw chicken patties with addition of CP and OMP during storage. As shown in Table 2, pH values of raw chicken patties without adding antioxidants (CTL) were higher (p<0.05) than those with addition of CP and OMP. They were also higher than those of REF (0.1% AA). Addition of CP and OMP could decrease pH, regardless of the addition level. However, pH values were found to be similar up to day 3 of storage. They started to increase from 7 days of storage. Then, they became higher toward the end of the storage time. Increased pH values of raw chicken patties with increasing storage time might be partially due to the breakdown of proteins to amino acids or alkali compounds by bacteria during storage. Masniyom et al [33] have reported that pH values of air-stored sea bass are increased during storage. This might be partially due to the production of basic amino acids or amines by bacteria, which are released during protein degradation. These bacteria could use stored glucose. Therefore, products of amino acid decomposition were accumulated, resulting in higher pH values.

CIE L*, a*, b* color values of raw chicken patties with/without antioxidants are shown in Table 4. CIE color values were affected by the addition of cinnamon and oyster mushroom, especially by the addition of 0.2% CP (C2M1, C2M2). Lightness (L*) values of control chicken patty samples were higher than those of treatments (p<0.05). However, these values remained stable up to 14 days during storage. The addition of 0.2% CP decreased L* value (lightness), but increased a* value (redness) and b* value (yellowness) due to original colors of CP and OMP. These results indicate that addition of CP and OMP alone or in combination might improve color values of chicken patties. Gahruie et al [34] observed natural extracts of cinnamon, shirazi, and rosemary with potential to improve color stabilities of frozen beef burger. These materials were able to control lightness and redness values. A similar study [35] has shown that the addition of spices of *Syzygium aromaticum*, *Cinnamomum cassia*, *Origanum vulgare*, and *Brassica nigra* to raw chicken meat can result in higher a* values, which indicated by intense red color due to spice carotenoids.

### Volatile basic nitrogen value

Byun et al [36] have described that VBN contents in meat are increased due to deamination of amino acids to ammonia during storage. VBN can be used to examine decomposition of fresh meat and poultry products. Volatile compounds such as (CH_3_)_3_NH (trimethylamine), (CH_3_)_2_NH (dimethylamine or DMA), and NH_3_ (ammonia) are products of microbial degradation. They are known as total volatile basic-nitrogen (TVB-N). Hence, TVB-N level is a potential indicator of fish spoilage [37]. As shown in Table 5, VBN values of raw chicken patties were increased in all treatments with increasing storage period. VBN values reached up to 14.64 mg N/100 g at day 14 of storage. Byun et al [36] reported that total VBN values are 20 and 30 mg N/100 g for beef and pork, respectively. Another study conducted by Balamatsia et al [38] found that chicken meat stored in air have acceptable VBN value of 40 mg N/100 g in a refrigerator. In the present study, chicken patties added with AA (REF) had lower VBN values than those treatments (p<0.05). Although no difference in VBN value was found among treatments (p>0.05), VBN values were still lower than those of CTL (no treatment with powder), indicating that combination of CP and OMP might be effective in retarding protein deterioration. In this study, lower VBN values of meat samples might be partially due to antimicrobial activities of CP and OMP. These results have been suggested by Gupta et al [39] who reported that cinamaldehyde compound on cinnamon bark is highly electro-negative, which is associated with biological processes in electron transfer by reacting with nitrogen-containing components, e.g., proteins and nucleic acids, therefore inhibiting the growth of microorganisms.

### Microbial counts

As shown in Table 5, both TPC and *Enterobacteriaceae* were increased rapidly from day 7 throughout the end of storage time. Chicken patties containing C2M1 and C2M2 showed lower TPCs than other treatments, while *Enterobacteriaceae* counts were lower in all treatments added with natural antioxidant. In this study, addition of CP up to 0.2% (C2) resulted in better antimicrobial activity regardless of OMP addition. These results indicate that the combination of CP and OMP can inhibit the growth of pathogen that partially due to antimicrobial activity of those materials. Hoque et al [40] studied the antimicrobial activity of cinnamon essential oil on ground chicken meat. They found that cinnamon inhibited microbial pathogens such as *Pseudomonas*, *L. monocytogenes*, and *Salmonella*. Iwolakun et al [41] have detected phenolics and terpenoids in oyster mushroom. Those constituents might have potential as antimicrobial agents. In addition, Shan et al [42] reported that total phenolics might be significantly associated with antibacterial activity. Extracts from medicinal herbs which contain high levels of phenolic compounds possess strong antibacterial activities. Thus, medicinal herbs might be potential sources of antioxidants that can help fight against foodborne pathogens.

### 2-Thiobarbituric acid reactive substances

**Figure 1:**
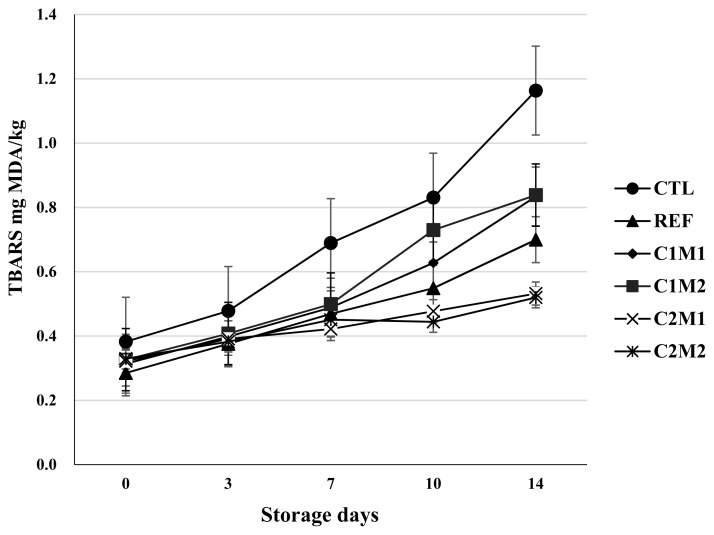
Thiobarbituric acid reactive substances(TBARS) values of chicken patties with various levels of cinnamon and oyster mushroom powder during storage. Treatments: CTL = control (without antioxidants); REF = patties mixed with 0.1% ascorbic acid; C1M1 = patties mixed with 0.1% cinnamon powder; 0.1% oyster mushroom powder, C1M2 = patties mixed with 0.1% cinnamon powder; 0.2% oyster mushroom powder, C2M1 = patties mixed with 0.2% cinnamon powder; 0.1% oyster mushroom powder and C2M2 = patties mixed with 0.2% cinnamon powder; 0.2% oyster mushroom powder.

Since there was an interaction between treatment and storage time, data were separated out by treatment within a storage time or by storage time within a treatment. As shown in Figure 1, TBARS values of all treatments were increased with increasing storage time. However, they were decreased with the addition of CP and OMP. Generally, TBARS values might be correlated with the acceptable level for sensory panelists to consume meat products [43]. CTL had the highest TBARS value and REF showed the lowest TBARS value among treatments, while C2M1 and C2M1 had lower TBARS than 0.1% CP at the initial storage (p<0.05). Interestingly, C2M1 and C2M2 were found the lowest TBARS values (comparable to that of AA/REF). These results indicated that increased level of CP was more effective in inhibiting lipid oxidation than those without CP, regardless of the addition of OMP. These results were consistent with those of El-Alim et al [44] who found that dried spices of cinnamon, peppermint, cloves, nutmeg, marjoram, curry, and caraway could inhibit lipid oxidation of raw minced chicken meat by reducing the TBARS level after 6 months frozen storage to two to three times lower than the control (without any spices). In the present study, adding CP and OMP was effective in inhibiting lipid oxidation and microbial count in ground chicken patties during storage. Arbaayah and Umi [45] have reported that phenolic compounds such as phenolic acids and tannins are major components of the antioxidant system in plants and mushrooms.

## CONCLUSION

Powder from cinnamon bark was proven to be an antioxidant activity. The highest activity was obtained from CP extracted with ethanol extraction 80%. Raw chicken patties made with CP and OMP had lower pH value, TBA, VBN, and microbial counts of total bacteria and *Enterobacteriaceae* as compared to those in the control during refrigerated storage. Redness (a*) values tended to be stable up to 14 days. However, lightness (L*) was decreased with increasing storage time. This study suggests that the addition of CP and OMP into raw chicken patties improved their shelf-life during refrigerated storage. Adding 0.2% of CP to chicken patties was more effective than others in terms of antioxidant and antimicrobial activities, regardless of the OMP levels added.

## Figures and Tables

**Table 1:** Formulation of raw chicken patties with addition of different levels of cinnamon and oyster mushroom powders

Ingredients (%)	Treatments^1)^					

	CTL	REF	C1M1	C1M2	C2M1	C2M2
Chicken breast	98.16	98.16	98.16	98.16	98.16	98.16
Salt	1.84	1.84	1.84	1.84	1.84	1.84
Ascorbic acid (AA)	0.00	0.10	0.00	0.00	0.00	0.00
Cinnamon	0.00	0.00	0.10	0.10	0.20	0.20
Oyster mushroom	0.00	0.00	0.10	0.20	0.10	0.20
Total	100.0	100.1	100.2	100.3	100.3	100.4

1) CTL =control (without antioxidants); REF = patties mixed with 0.1% ascorbic acid; C1M1 = patties mixed with 0.1% cinnamon powder, 0.1% oyster mushroom powder; C1M2 = patties mixed with 0.1% cinnamon powder, 0.2% oyster mushroom powder; C2M1 = patties mixed with 0.2% cinnamon powder, 0.1% oyster mushroom powder; C2M2 = patties mixed with 0.2% cinnamon powder, 0.2% oyster mushroom powder.

**Table 2:** Antioxidant activities of cinnamon powder in various concentrations with different solvent

Parameters	Treatments^1)^	Concentration (%)				

		0	0.1	0.25	0.5	1.0
DPPH-RSA (%)	AA	0.00^b^^A^	92.8^a^^A^	93.3^a^^A^	93.4^a^^A^	94.8^a^^A^
	CP	0.00^b^^A^	44.5^a^^B^	49.2^a^^B^	44.9^a^^B^	53.1^a^^B^
	CWE	0.00^b^^A^	39.9^a^^C^	35.6^a^^BC^	36.1^a^^BC^	39.5^a^^C^
	CEE 50	0.00^b^^A^	25.6^ab^^D^	21.4^b^^C^	21.5^b^^C^	36.8^a^^C^
	CEE 80	0.00^b^^A^	42.1^a^^BC^	50.1^a^^B^	58.3^a^^B^	68.8^a^^B^
	CEE 100	0.00^b^^A^	43.1^a^^B^	40.1^a^^B^	37.2^a^^BC^	37.5^a^^C^
Iron chelating ability (%)	EDTA	0.00^b^^A^	95.8^a^^A^	97.9^a^^A^	98.3^a^^A^	98.6^a^^A^
	CP	0.00^d^^A^	6.77^c^^E^	16.9^c^^C^	18.8^b^^C^	22.5^a^^D^
	CWE	0.00^d^^A^	21.2^c^^B^	37.2^b^^B^	62.7^ab^^B^	83.1^a^^B^
	CEE 50	0.00^d^^A^	19.1^c^^D^	27.7^b^^BC^	25.7^a^^C^	39.5^a^^D^
	CEE 80	0.00^d^^A^	23.5^c^^B^	26.4^b^^C^	26.3^ab^^C^	33.9^a^^D^
	CEE 100	0.00^d^^A^	15.9^c^^C^	19.7^b^^C^	25.3^ab^^C^	53.2^a^^C^
Reducing power (%)	AA	0.00^b^^A^	1.43^a^^A^	1.44^a^^A^	1.49^a^^A^	1.89^a^^A^
	CP	0.00^d^^A^	0.27^c^^B^	0.24^c^^C^	0.68^b^^C^	1.02^a^^C^
	CWE	0.00^d^^A^	0.29^c^^B^	0.40^c^^B^	0.59^ab^^B^	1.01^a^^C^
	CEE 50	0.00^d^^A^	0.63^c^^B^	1.04^b^^B^	1.20^ab^^B^	1.42^a^^B^
	CEE 80	0.00^d^^A^	0.55^c^^B^	1.24^b^^B^	1.38^ab^^B^	1.59^a^^B^
	CEE 100	0.00^d^^A^	0.45^c^^B^	0.98^b^^B^	1.17^a^^B^	1.34^a^^B^

DPPH-RSA, 2,2-diphenyl-1picrylhydrazyl-radical scavenging activity.

1) AA, ascorbic acid; CP, cinnamon powder; CWE, cinnamon water extract; CEE 50, cinnamon ethanol (50%) extract powder; CEE 80, cinnamon ethanol (80%) extracted powder: CEE 100, cinnamon ethanol (100%) extracted powder; EDTA, ethylenediamine tetraacetic acid.

a,b,c,d Means with different superscript letters in the same row indicate differences at p<0.05.

A,B,C,D Means with different superscript letters in the same column indicate differences at p<0.05.

**Table 3:** Total phenolic compounds (mg GAE/g) of cinnamon powder extracted with different solvents

Treatments^1)^	Cinnamon powder (CP)	CWE	CEE 50	CEE 80	CEE 100
Total phenolic compounds (mg GAE/g)	11.77^b^	13.85^b^	18.26^a^	19.73^a^	17.93^a^

GAE, gallic acid equivalents.

1) CP, cinnamon powder; CWE, cinnamon water extract; CEE 50, cinnamon ethanol (50%) extract powder; CEE 80, cinnamon ethanol (80%) extracted powder: CEE 100, cinnamon ethanol (100%) extracted powder.

a,b Means with different superscript letters in the same column indicate differences at p<0.05.

**Table 4:** pH and color values of chicken patties as affected by different levels of cinnamon and oyster mushroom powders

Treatments^1)^		Parameter			

		pH	Color L^*^	Color a^*^	Color b^*^
CTL	Mean	6.10^c^	49.12^a^	−0.53^c^	5.82^c^
	SD	0.13	0.81	0.66	0.64
REF	Mean	5.89^a^	48.41^ab^	−0.46^c^	5.82^c^
	SD	0.09	0.98	0.72	0.58
C1M1	Mean	6.00^b^	48.02^abc^	−0.28^b^	6.15^b^
	SD	0.10	1.12	0.76	0.75
C1M2	Mean	5.95^b^	47.55^bc^	−0.24^b^	6.38^ab^
	SD	0.12	1.05	0.71	0.71
C2M1	Mean	5.95^b^	46.93^c^	−0.11^ab^	6.51^a^
	SD	0.10	1.26	0.87	0.75
C2M2	Mean	5.95^b^	46.96^c^	−0.02^a^	6.63^a^
	SD	0.10	1.69	0.83	0.74
Storage days					
0	Mean	5.87^C^	48.13^A^	1.12^A^	7.12^A^
	SD	0.05	1.24	0.35	0.50
3	Mean	5.87^C^	48.47^A^	−0.51^B^	5.59^D^
	SD	0.04	1.66	0.31	0.51
7	Mean	6.02^B^	47.31^A^	−0.50^B^	5.67^D^
	SD	0.05	1.27	0.23	0.30
10	Mean	5.98^B^	47.54^A^	−0.70^C^	5.98^C^
	SD	0.09	1.05	0.17	0.41
14	Mean	6.12^A^	47.70^A^	−0.77^C^	6.73^B^
	SD	0.06	1.51	0.16	0.41

SD, standard deviation.

1) CTL =control (without antioxidants); REF = patties mixed with 0.1% ascorbic acid; C1M1 = patties mixed with 0.1% cinnamon powder, 0.1% oyster mushroom powder; C1M2 = patties mixed with 0.1% cinnamon powder, 0.2% oyster mushroom powder; C2M1 = patties mixed with 0.2% cinnamon powder, 0.1% oyster mushroom powder; C2M2 = patties mixed with 0.2% cinnamon powder, 0.2% oyster mushroom powder.

a,b,c Means with different superscripts among treatments are different (p<0.05).

A,B,C,D Means with different superscripts the same storage days are different (p<0.05).

**Table 5:** Volatile basic nitrogen (VBN), total plate counts (TPC), and *Enterobacteriaceae* (VRB) of chicken patties as affected by different levels of cinnamon and oyster mushroom powders

Treatments^1)^		Parameters		

		VBN (mg/100 g)	TPC (log cfu/g)	VRB (log cfu/g)
CTL	Mean	11.58^a^	4.20^a^	4.17^a^
	SD	2.95	0.79	0.75
REF	Mean	9.77^c^	3.94^bc^	3.76^b^
	SD	2.42	0.79	0.58
C1M1	Mean	10.57^b^	4.05^b^	3.82^b^
	SD	2.63	0.61	0.57
C1M2	Mean	10.52^b^	3.95^bc^	3.80^b^
	SD	2.83	0.77	0.65
C2M1	Mean	10.87^b^	3.88^c^	3.77^b^
	SD	3.16	0.72	0.52
C2M2	Mean	10.57^b^	3.90^c^	3.71^b^
	SD	2.87	0.64	0.62
Storage days				
0	Mean	6.63^C^	3.51^D^	3.25^E^
	SD	0.54	0.14	0.13
3	Mean	10.48^B^	3.43^D^	3.39^D^
	SD	1.32	0.24	0.14
7	Mean	10.69^B^	3.65^C^	3.56^C^
	SD	1.13	0.17	0.20
10	Mean	10.79^B^	4.08^B^	4.24^B^
	SD	1.01	0.19	0.28
14	Mean	14.64^A^	5.25^A^	4.76^A^
	SD	1.07	0.18	0.25

VBN, volatile basic nitrogen; TPC, total plate counts; VRB, violet red bile; SD, standard deviation.

1) CTL =control (without antioxidants); REF = patties mixed with 0.1% ascorbic acid; C1M1 = patties mixed with 0.1% cinnamon powder, 0.1% oyster mushroom powder; C1M2 = patties mixed with 0.1% cinnamon powder, 0.2% oyster mushroom powder; C2M1 = patties mixed with 0.2% cinnamon powder, 0.1% oyster mushroom powder; C2M2 = patties mixed with 0.2% cinnamon powder, 0.2% oyster mushroom powder.

a,b,c Means with different superscripts among treatments are different (p<0.05).

A,B,C,D,E Means with different superscripts among storage days are different (p<0.05).
